# A New Mechanical Mouth Opener for Dynamic Magnetic Resonance Imaging of the Temporomandibular Joint

**DOI:** 10.3390/jcm12155035

**Published:** 2023-07-31

**Authors:** Milica Jeremic Knezevic, Aleksandar Knezevic, Jasmina Boban, Aleksandra Maletin, Bojana Milekic, Daniela Djurovic Koprivica, Ivana Mijatov, Tatjana Puskar

**Affiliations:** 1 Faculty of Medicine Novi Sad, University of Novi Sad, 21000 Novi Sad, Serbia; aleksandar.knezevic@mf.uns.ac.rs (A.K.); jasmina.boban@mf.uns.ac.rs (J.B.); aleksandra.maletin@mf.uns.ac.rs (A.M.); bojana.milekic@mf.uns.ac.rs (B.M.); daniela.djurovic-koprivica@mf.uns.ac.rs (D.D.K.); ivana.mijatov@mf.uns.ac.rs (I.M.); tatjana.puskar@mf.uns.ac.rs (T.P.); 2 Medical Rehabilitation Clinic University Clinical Center of Vojvodina, 21000 Novi Sad, Serbia; 3 Center for Imaging Diagnostics, Institute for Oncology, 21208 Sremska Kamenica, Serbia; 4 Dentistry Clinic of Vojvodina, 21000 Novi Sad, Serbia; 5 University Clinical Center of Vojvodina, Clinic for Oral and Maxillofacial Surgery, Faculty of Medicine Novi Sad, University of Novi Sad, Department of Stomatology with Maxillofacial Surgery, 21000 Novi Sad, Serbia

**Keywords:** temporomandibular joint, magnetic resonance imaging, temporomandibular disorders, polymer materials, polymeric mouth opening device, dentistry, mechanical device

## Abstract

(1) Background: During the magnetic resonance imaging (MRI) of the temporomandibular joint (TMJ), it is necessary to scan the joints in the closed- and open-jaw position, as well as in the maximally open-jaw position. In order to examine both joints in these positions, an MRI compatible mouth opener is required, which allows the articular surfaces to maintain their position stably. (2) Methods: In this study, we included 200 patients aged 18 to 65, with various levels of clinical severity. The mouth opener is made of polymethyl methacrylate and used for dynamic imaging of TMJ. It is in the form of an arrow, with incisures on upper and lower surfaces 1 mm apart and these match possible variations in jaw opening. All the patients were scanned with mouth opener and, immediately after this scanning, with syringe (20 ccm) as a standard device used for mouth opening in clinical setting. (3) Results: A total of 200 MR examinations of TMJs were performed and the mechanical mouth opener was successfully applied without artifacts in all patients. The mouth opener device proved to be adequate in case of MRI of the TMJ for different ranges of mouth opening with the proper protocol for provoked imaging, because the incisures are located at a distance of 1 mm and no objective artifacts were observed in any examination that degraded the diagnostic quality of the examination. (4) Conclusions: The design of the acrylate mouth opener is precisely defined, and it has a purpose in the MRI diagnosis of TMJ disorders.

## 1. Introduction

The temporomandibular joint (TMJ) is one of the most complex and frequently used joints in the body, and magnetic resonance imaging (MRI) has been considered a golden standard in diagnosing the temporomandibular joint disorders (TMDs), especially in diagnosing the internal derangement, disc disclocations, retrodiscal tissue, and bone marrow [1].

The orofacial (stomatognathic) system is a complex adaptive system that comprises temporomandibular joints, teeth, periodontium, bone, intra- and perioral soft tissues, masticatory and accessory muscles, and the peripheral and central nervous system that integrates, coordinates, and controls associated orofacial movements. The orofacial system is responsible for the functions of food intake, articulation of sounds, breathing, and expression of emotions [2]. If any component of the orofacial system is damaged, some of these functions will be impaired.

TMJ is part of the orofacial system, which belongs to the group of condylar synovial diarthroses, which enables it to have a wide range of motion in all three planes [3]. The basic anatomical components of the TMJ are condyle or head of the mandible, mandibular fossa, articular disc, joint capsule, joint ligaments, and lateral pterygoid muscle [4]. Diseases of the TMJ represent a larger group of disorders that include the TMJ, masticatory muscles, tendons, and/or surrounding structures [5] and are called TMDs, occurring mostly in younger and middle-aged women. The most significant clinical signs and symptoms of TMDs are pain in the area of the masseter muscle, the TMJ region, the temporal muscle region, the ear region, limited opening of the mouth, sounds in the joint, such as clicks and crepitations, and headaches [6]. TMJs relate to all movements of the lower jaw including speech, mastication—opening and closing mouth, with a frequency of approximately 2000 times a day [7]. Due to the high frequency, the discovery of the lesion in TMJs has a great influence on daily life. In a systematic review by Bitiniene et al., it was found that there is a direct correlation between TMDs and lower quality of life [8]. Similar results were found in the study by Pigozzi et al. where they found that there is a direct connection between quality of life and pain intensity and disability reported by TMDs patients [9]. Psychosocial factors could also be an etiological factor for TMDs. In a systematic review by Minervini et al., they showed that the state of uncertainty in which people lived during pandemics could also contribute to TMDs, especially through stress-related muscle hyperactivity and aggravation of bruxism [10]. They suggested that clinicians must assess this new issue of COVID-19-related anxiety and stress, and, if TMDs are related to this situation, intervene with appropriate therapies, which should be carried out by multidisciplinary teams consisting of dentists, physicians, and psychologists [11]. Ginszt et al. concluded that significant changes in electromyographic patterns due to medical mask-wearing were observed in patients with muscular dysfunctions compared to healthy controls [12]. 

It Is important to emphasize the relationship between poor body posture and the incidence of TMDs. However, commonly, it is not possible to establish whether TMDs are a cause or a result of body posture deviations. Patients with TMDs present with an overload in cervical muscles due to increased activity of the masticatory muscles to compensate for the TMJ disorder. Such an overload can produce mandibular and spinal deviations and cervical hyperlordosis, due to shoulder elevation and head protrusion in patients with TMDs [13]. Walczyńska-Dragon et al. also concluded that most subjects reported improvement and/or disappearance of cervical spine pain and TMDs symptoms after applying treatment with an occlusal splint [14]. In contrast to previous authors, Raya et al. presented similar prevalence of alteration in the craniocervical position in women with and without TMDs, so the actual role of TMDs in the cervical region posture is still a matter of debate [15]. It has also been noticed that closing and opening of the eyes affected the electromyographic (EMG) patterns of the masticatory and cervical muscles in myopic subjects [16].

Epidemiological studies show that 50–75% of the general population have some sign of dysfunction in the orofacial system during their lifetime, and the most common reason for being referred to a physician is pain [17]. Some authors estimate that the prevalence of TMDs is 5–12% in the general population, thus representing the second most frequent location of musculoskeletal pain (after low back pain) and a very frequent cause of absenteeism [18]. A recent report of Valesan et al. in 2021 estimated that the prevalence of TMD was 31% for adults and 11% for children and adolescents [19].

Internal derangement is present in more than 65% of patients with clinical symptoms of the TMJ. The most frequent finding in internal derangement is an anterior dislocation of the disc with reduction. Disc displacement may be caused by trauma, hypercontraction of lateral pterygoid muscles, laxity of ligaments, bruxism, or synovial fluid alteration [20,21]. Moreover, internal derangement is a risk factor for developing osteoarthritis with remodeling of the articulating surfaces—condyle and the fossa mandibularis [20].

In the past, clinical examinations and specific protocols were the only possible way to diagnose TMDs. With the development of modern technologies, through arthrography (1944), arthrotomography (1970), and computerized tomography (CT) and MRI in 1984, new valuable knowledge was gained [21]. Today, MRI, as an imaging method, represents the “gold standard” for establishing the position of the articular disc, the presence of synovial fluid in the TMJ [22], the condition of the retrodiscal tissue, and the bone marrow signal of the mandibular canal. Moreover, MRI is a non-invasive, non-ionizing, and non-contrast method for diagnosing internal disorders of the TMJ [20,23,24]. Today, MRI is routinely used in patients with suspected or known TMDs.

The earliest data concerning problems related to the TMJ date from the fifth century BC, when Hippocrates described a manual method of fixing dislocations of the mandible, called “mandibular fixation”. In 1934, a significant contribution to this field of research was made by an otorhinolaryngologist James Costen, who described a set of symptoms which were related to the TMJ, ears, and sinuses in patients with reduced vertical dimension of occlusion in jaw proportions, later known as Costen’s syndrome [25]. At that time, due to the poorer knowledge of anatomy, as well as functional anatomy of the TMJ, the etiology, the diagnostics, and, consequently, the treatment of these disorders was rather difficult.

The first image in MRI of TMJ was taken in 1984. In the first years since the introduction of MRI into medicine, static imaging of the TMJ was performed, i.e., imaging in the closed mouth position. It was only after the introduction of new techniques, protocols, and sequences that dynamic or kinematic imaging of the TMJ became possible, i.e., imaging of the TMJ when opening the mouth. Then, there was a need for a tool that would allow the lower jaw, the mandible, to be stable during the TMJ imaging in the position of the mouth as wide as possible. A problem that can arise if the mandible moves during imaging is the appearance of artifacts or blurring, which degrades the quality of the image and questions the diagnostic accuracy of the examination. 

The imaging of TMJ by MRI requires a dedicated patient posturing. Adequate position of the mandible is essential both for soft tissue and osseous imaging. During MRI of TMJ, it is necessary to obtain images in both positions of the closed jaw position (maximal intercuspal position of the lower jaw) and in the position of the maximally open jaw. Maximally opened jaw position is essential for evaluating condyle position and position and shape of the disc. One of the biggest challenges and problems that exist during MRI of the TMJ is scanning during the opening of the mouth, since for a high-quality and diagnostically valid image, it is necessary for the patient to be perfectly still in the open-mouth position during the several minutes that the examination of both TMJs takes. It is necessary for the patient to maintain the same position for 10–15 min, which is very difficult. In this position, the lower jaw moves, because very often patients who come for imaging of the TMJ suffer pain in the TMJ and/or in the surrounding muscles, predominantly the masseter muscle and medial pterygoid muscle. If the maximally opened position of the jaw is suboptimally accomplished, the upper and lower joint space of the TMJ and articular disc cannot be reliably examined, and the proposed diagnosis is not accurate. However, a number of patients are not able to remain adequately positioned during the examination by MRI, so prefabricated mouth openers (applicators) are commonly used to maintain the proper position of opened jaw.

Several mouth openers have been proposed in both journals and books. Vogl et al. presented an incremental hydraulic jaw opener for MR imaging of the TMJ, which enables reproducible and stable positions of TMJ by modified gradient pulse sequences. They determined the condyle-disk complex at each open-mouth position, with special attention to maximum mouth open positions by jaw opener [26]. A commercially available mouth opener, Medrad Burnett TMJ-200 Bi-Directional Device (GE Healthcare, Pittsburgh, Pennsylvania, USA), is also for dynamic assessment of TMJ by MRI, and it could be expensive for some institutions [27].

Szopinski and Regulski presented a simple graded bite block for dynamic MRI of the TMJ [28]. On the upper part, the bite block has a groove for the incisal edges of the upper incisors while on the lower part, there are steps with bite surfaces for the incisal edges of the lower incisors. A 3D printer is necessary for manufacturing this mouth opener and authors used a 1.5 T MRI scanner in the study.

Dynamic and pseudo-dynamic TMJ MRI techniques was introduced by some researchers in 1987 [1]. According to Kamel et al., the dynamic MRI was evaluated as a contemporary diagnostic tool for examination of TMJ by comparing it to the static MRI by clinical findings and ability to diagnose various types of disc displacement, as well as for the assessment of the quality of the image by visibility of the important anatomical TMJ structures such as disc and condyle, and the presence of artifact [29]. Kamel et al. concluded that dynamic MRI facilitated visualization of the articular disc during the entire pathway of jaw opening and closing as well as the dynamics of internal derangement and assessment of disc condyle relationship in a short time compared to static MRI. Moreover, it is better at evaluating disc mobility than static MRI.

The purpose of this study was to design and present a simple MRI-compatible device used for maintenance of the open-jaw position during dynamic MRI examination of the TMJ, designed to be used for various severity of the clinical condition, enabling accurate and reliable results that are applicable in the everyday practice of dentists and radiologists. Thus, research in the field of diagnostics of TMDs and making a new mouth opener for examination TMJ by MRI play a crucial role in TMJ health and general health.

## 2. Materials and Methods

A total of 200 subjects were included in this institutional ethical board-approved study, within a two-year period. The study was conducted in accordance with the Declaration of Helsinki and approved by the Institutional Review Board (or Ethics Committee) of Faculty of Medicine Novi Sad, University of Novi Sad (approval number: 01-39/14). All participants signed informed consent for entering the study.

The mouth opener stabilized the position of the lower jaw in maximum mouth position adequately in all (100%) patients. Only one patient (2 TMJs) was excluded from the study, due to missing incisors, making him physically unable to hold the mouth opener.

**Figure 1:**
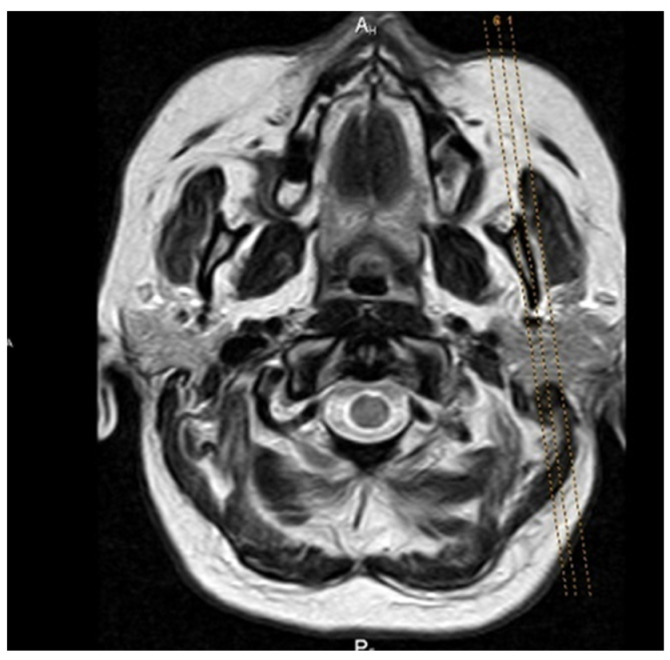
The position of parasagittal sections of the TMJ following the plane of the body of the mandible.

All patients underwent MRI on a 3 T unit (Siemens Trio Tim, Erlangen, Germany), with standard MR protocol for brain and dedicated imaging of TMJ: proton density sequence was used, time of repetition 1850 ms, time of echo 15 ms, field of view 13 cm, matrix size 256 × 256, and slice thickness 2 mm (Figure 1). The MRI was performed after clinical examination, using TMJ surface coil, with parasagittal and coronal tomograms of both condyles. All the patients were scanned with a mouth opener and, immediately after this scanning, with a syringe (20 ccm) as a standard device used for mouth opening in a clinical setting. 

The inclusion criteria were as follows: patients over 18, the presence of upper and lower (to be able to hold the applicator in the mouth) incisors, comprehension of the Serbian language (necessary for filling in the forms), and willingness to participate in this study.

The exclusion criteria were contraindications for MRI, technically inadequate MRI tomograms, pregnancy, presence of systemic disease involving TMJ and masticatory muscles, brain tumors, multiple sclerosis, metastatic disease, infectious and inflammatory central nervous system diseases, deprived consciousness, psychiatric disorders, and recent facial trauma. The presence of these conditions was verified on the screening FLAIR image of the head evaluated by the neuroradiologist (J.B.) and also after careful investigation of patient’s history available in the medical documentation. 

One experienced radiologist in head and neck imaging with over 15 years of work in the field of maxillofacial radiology and neuroradiology examined TMJs by MRI (J.B.). The second rater was dental medicine doctor, specialist of prosthodontics, with over 15 years of experience and special focus on TMDs (M.J.K.). If two raters made a different diagnosis, final diagnosis was established by consensus.

**Figure 2:**
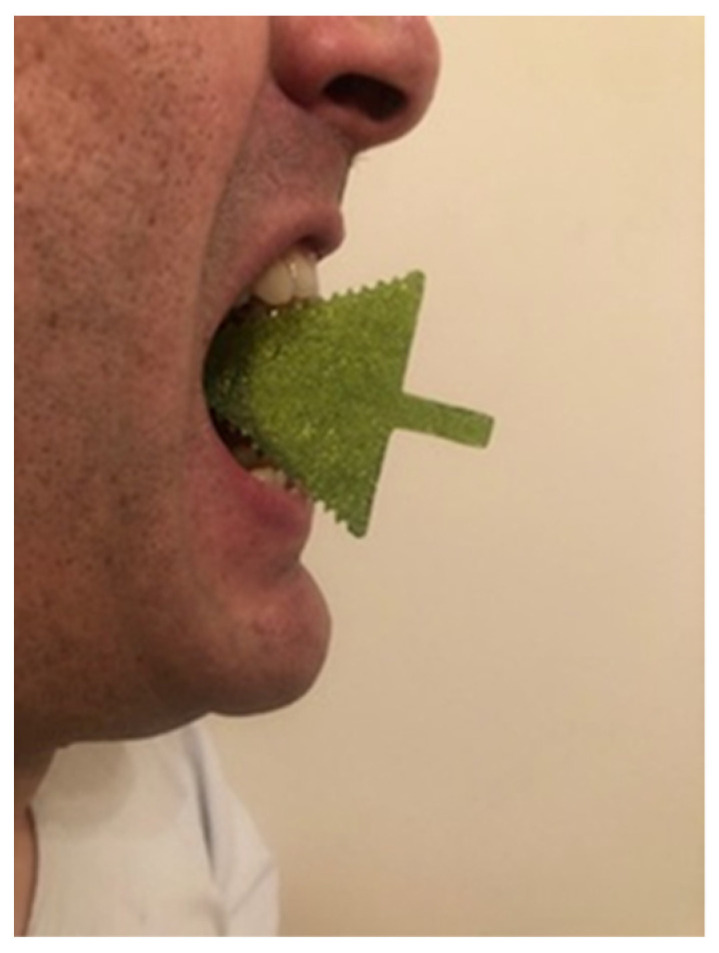
Position of the polymethyl methacrylate (PMMA) mouth opener in the patients’ mouth during MRI examination.

**Figure 3:**
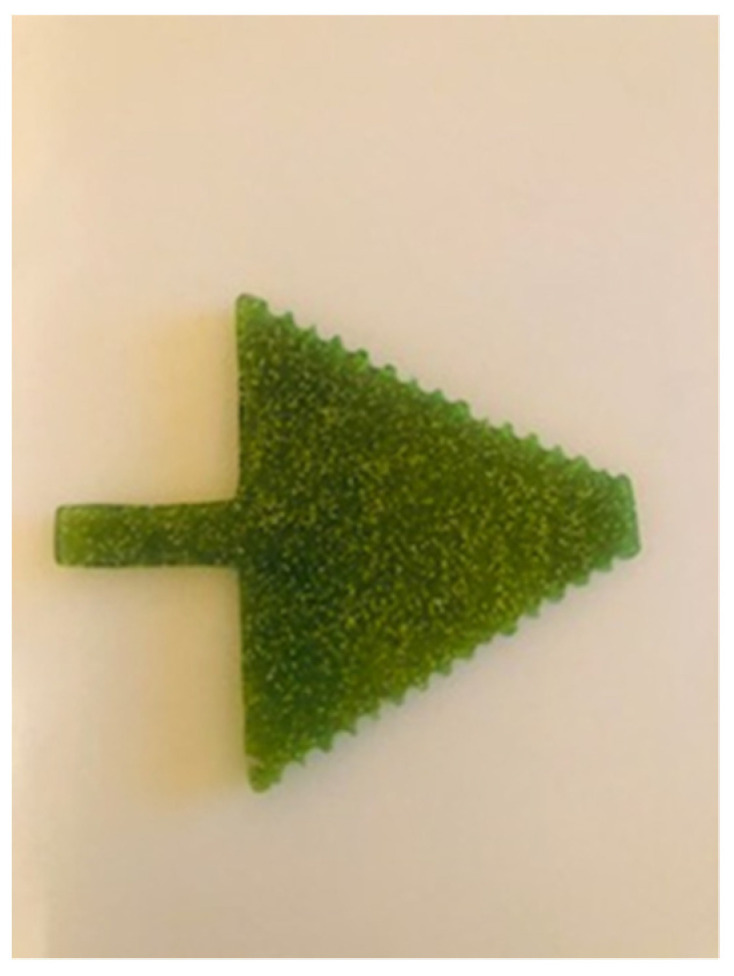
Mouth opener in the form of arrow, incisures are located at a distance of 1 mm.

**Figure 4:**
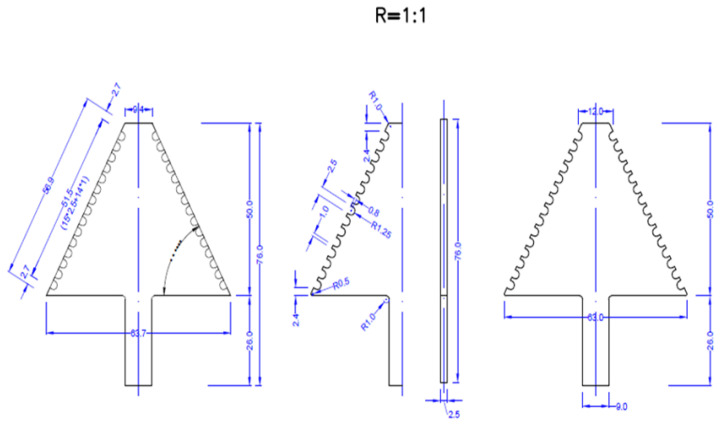
Technical drawing of the mouth opener from all directions. All dimensions are given in millimeters.

A polymeric mouth opener made of polymethyl methacrylate (PMMA) was used for dynamic imaging of the TMJ. The device is in the form of an arrow, with incisures on upper and lower surfaces (places to put incisors inside) 1 mm apart; these match possible variations in jaw opening (Figure 2, Figure 3 and Figure 4). The thickness of the device is 2.5 mm. While inside the MRI unit, after having completed sequences performed with closed jaw, the patient placed the applicator in their mouth, according to instructions given by the radiographer. 

**Figure 5:**
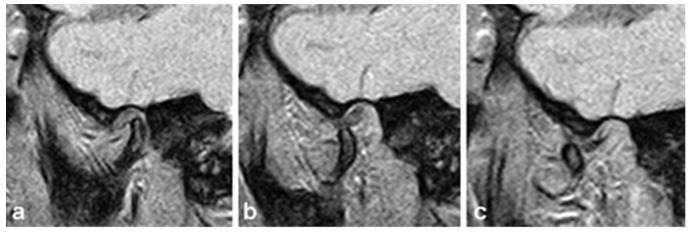
Sagittal view of TMJ on MRI. (**a**) Closed mouth position. (**b**) Semi-opened mouth position, 25 mm distance between upper and lower incisors. (**c**) Maximum mouth opening position.

The basic protocol for MRI of TMJ is comprised of the closed and maximally open jaw positions. In order to record both TMJs in the maximally open jaw position, a mouth opener is required, which enables the articular surfaces to maintain stable position during one MRI sequence (Figure 5). In a closed-mouth position, the examination was performed without the opening device. 

### 2.1. Testing Protocol for Artifacts

The propound mouth opener is applied as a clinical prototype for the purpose of examination of the TMJ by MRI.

The overall quality of the obtained tomograms with the mouth opener was evaluated according to the principle of binary classification: acceptable/unacceptable.

There are four basic types of artifacts on tomograms that degrade the diagnostic quality of the image (Table 1):

1. Signal-to-noise-ratio (SNR), which is further divided into three basic groups of artifacts (patient-dependent, hardware-dependent): edge artifacts, flow artifacts, and aliasing artifacts.
2. Artifacts related to the property of matter to be magnetized in a magnetic field (primarily refers to ferromagnetic and paramagnetic materials that cause defects in the voxel grid of sections).
3. The intensity of the signal depends on the device itself.
4. Homogeneity of the signal, which depends on the homogeneity of the MR device field.

An acceptable image was considered to have a total score of 0–4 for the presence of artifacts, while a score higher than 4 (5–12) was marked as a diagnostically unacceptable TMJ image.

### 2.2. Statistical Analysis

The statistical analysis was carried out using SPSS ver. 21.0 (Statistical Package for the Social Sciences, Chicago, IL, USA). The means and standard deviations of the variables were determined with descriptive statistical analyses.

The sample size was calculated by GPower (G*Power, Version 3.1.9.6, Heinrich Heine Universität, Düsseldorf, Germany) [30]. The number of participants needed to achieve a statistical power of 0.80 for medium-size effect and an α error probability of 0.05 was 145. 

In order to assess reliability, intraclass correlation coefficient two-way random model (ICC) was used. The ICC values < 0.4 were interpreted as poor, 0.4–0.59 as fair, 0.6–0.75 as good and >0.75 as excellent reliability [31]. *p* values < 0.05 were considered statistically significant and <0.01 as highly statistically significant.

## 3. Results

Patients did not report any significant discomfort during MR examination of TMJ.

On the MRI, no artifacts were detected derived by the mouth opener (MR compatible) and diagnosis could be established by two independent examiners.

In Table 2, based on MR examination, two experienced raters separately diagnosed various levels of conditions of TMD. The largest number of TMJs were without pathological changes, 198 of them (49.5%). Anterior disc dislocation with reduction was found in 46 TMJs (11.5%), followed by anterior disc dislocation without reduction (18 TMJs (4.5%)), posterior dislocation 4 TMJs (1), osteoarthritis (OA) in (100 TMJs (25%)). The combination of disc dislocation with reduction and OA was found in 20 TMJs (5%), disc dislocation without reduction and OA in 6 TMJs (1.5%), while the combination of posterior dislocation and OA was detected in 8 TMJs (2%).

During the TMJ MRI examination with the proper protocol for provoked imaging with the described mouth opener, no artifacts were observed in any examination that degraded the diagnostic quality of the examination, given the fact that the opener is made of MR-biocompatible, bioinert material. 

The mouth opener proved to be adequate due to the possibility of examining the TMJ by MRI, with different ranges of mouth opening, because the incisures on arrow are located at a distance of 1 mm. It is very important to examine the TMJ with a dynamic approach in the maximally open-mouth position, because certain TMJ diseases, such asdislocations of the articular disc, hypermobility, or subluxation of the condyle, can only be diagnosed in both mouth positions, closed-mouth position and maximally open-mouth position.

In Table 3 and Table 4, we present the results on interrater reliability between the experienced radiologist and the clinical dental medicine doctor and also intermethod reliability, with a syringe used as a standard device for mouth opening in the clinical setting of TMJ imaging. Intraclass correlation coefficient demonstrated excellent reliability between raters and methods of their ratings across subjects.

## 4. Discussion

The goal of designing and manufacturing an acrylate mouth opener is to obtain a standardized device for determining the condition of patients with both emergency [32] and long-term problems of the TMJ and therefore provide the adequate treatment after MRI examination. The presented mouth opener is an original clinical device for examination of the TMJ by MRI. The results of this study show that accurate diagnoses of the TMDs without artifacts and the evaluation of the TMJ by MRI can be performed by polymethyl methacrylate mouth opener.

MRI is a crucial tool in the diagnostics of TMDs because it has ability to show articular discus without using ionizing radiation. In a diagnostic algorithm, the MRI uses no i.v. administration of contrast material and therefore presents a noninvasive method, and also provides information about the position, shape, and length of the articular disc, quantitative data about the presence of synovial fluid in TMJ, and qualitative data about the state of retrodiscal tissue and bone marrow. MRI is a powerful and noninvasive tool for the imaging and understanding of TMD; therefore, it is a common method used to diagnose TMDs [33]. Moreover, MRI is the best imaging modality for the evaluation of intra-articular processes in TMJ and given the high MRI contrast resolution of the soft tissues, it is currently the gold standard for diagnosing disc disorders [1,20,34]. Our study was performed on a 3 T MRI unit. High-resolution of 3 T magnetic field strengths have better overall image quality, better visibility, and delineation of both TMJ disc and part near to the condylar neck of pterygoid muscle. Due to higher signal-to-noise ratios, 3 T MRI magnets have the advantage of depicting improved anatomic and pathologic details of the TMJ compared with 1.5 T. Our idea was to compare the quality of images using the new solution (mouth opener) and the standard device used in the clinical setting (syringe), so we decided to evaluate the images acquired on a higher field strength which is also more susceptible to the artifacts.

Many clinical and scientific centers dealing with the diagnosis of TMJ diseases, and in the absence of the above-mentioned mouth openers, use plastic syringes of a certain volume (20-cc syringe—diameter approximately 20 mm) that are placed in patients’ mouths in a horizontal position during the TMJ MR examination. In this way, it is not possible to ensure a fixed position of the mandible during imaging, and it is very uncomfortable for the patient, given that the syringe has only one diameter and the position of fully opened jaw is not adequately achieved in all patients. An additional problem is that the use of a syringe allows the opening of the mouth to the same extent for each patient, that is, individualized access to the examination is lost.

The principles of all the above-mentioned mouth openers is to open and close the mouth in exactly defined steps and to provide motion-free images [35].

When choosing the material for making this device, it should be ensured that it is compatible with the strong MR field, it should be made of materials that do not possess ferromagnetic or paramagnetic properties, so as not to endanger the patient or degrade the quality of the examination due to the presence of artifacts. For this reason, polymethylmethacrylate is the material of choice for mouth openers used in TMJ imaging using MRI.

These materials are the most commonly used materials in some fields of dentistry, such as prosthetics and orthodontics, and they also have a well-documented history of use as biomaterials in the manufacturing of different types of dental appliances and devices [36]. Many positive properties of PMMA, such as low density, aesthetics, cost-effectiveness, ease of manipulation, and efficient mechanical and biological features, make it a suitable and popular biomaterial for these dental applications [37].

The field of examination of the TMDs is widespread, and there is a large amount of research on this topic [20,38,39], but so far, no tool has been designed that will not burden the budget of the institution where disease diagnosis and further research is carried out. It is necessary to approach it very precisely, given that pain in the TMJ region is the second most common musculoskeletal disorder after chronic low back pain, and, along with the improvement of diagnostic protocols, it would be wise to consider forming teams consisting of dentists, radiologists, psychiatrists, neurologists, and orofacial surgeons in order to make an appropriate choice of therapy and monitoring of the patients with TMDs [40,41].

Additionally, this mouth opener represents a comfortable solution for the patient and a safe device for MRI scanning. The patient enters the MRI unit with the mouth opener in his hand. During the MRI examination of the TMJ, patient puts the mouth opener inside his mouth to achieve the maximum opening position (the incisures are at 1 mm so every patient can achieve maximum opening with maximum personal comfort), as guided by the technologist: after standard TMJ imaging in the closed-mouth position is finished (maximum intercuspal position), the patient bites the mouth opener with his/her upper and lower incisors in the individual maximum open mouth position, and keeps it like that for 3–4 min (while the sequence is acquired). It is comfortable for the patient, because the incisures at the sides of the mouth opener keep the mandible in the stable position during sequence acquisition. Compared to the standardly used device (syringe), there was excellent intermethod reliability in the terms of artifacts, with almost perfect matching. Given the greater comfort and non-inferiority in the terms of image quality that is achieved with the use of dedicated mouth opener, it seems reasonable to recommend it for use in clinical practice instead of the syringe.

Interrater analysis between experienced radiologist and clinical dental medicine doctor with special focus in TMD showed excellent reliability of this device in terms of generating artifacts. Both readers marked most of the images obtained with the mouth opener as “without any artifacts”; the lowest interrater reliability was observed in rating susceptibility and signal intensity (ICC 0.818, and 0.835, respectively). However, artifacts in these terms are acquired not only due to the device used for mouth opening but also due to metallic implants (many of them in the region of maxilla and mandible) as well due to subtle field inhomogeneities. The fact that there was excellent matching between two readers makes the recommendation of this mouth opener for use in clinical practice sensible and reliable. In our opinion, it is important to make the images readable for clinical doctors in everyday practice, not only for dedicated radiologists. The design of the acrylate mechanical mouth opener is accurately defined and has a purpose in the imaging of the TMJ using MRI and making a diagnosis of TMJ disease. Hence, the propound mouth opener is applied as a clinical prototype for the purpose of examination of the TMJ by MRI.

The TMJ is anatomically, embryologically, and physiologically a complex structure, functionally tightly connected to the rest of the craniomandibular complex. Contemporary imaging modalities, if used properly and according to adequate clinical implications and criteria, are able to depict different pathological processes and play a crucial role in establishing the right diagnosis and monitoring therapeutic effect. The key to the right diagnosis, however, still lies in thorough familiarity with the TMJ developmental and functional anatomy, as well as with the TMJ dysfunction related to the jaws, teeth, and cranial base [42].

The final but not neglectable advantage of our mechanical mouth opener is low production cost.

## 5. Conclusions

A mechanical mouth opener can be safely and simply used during dynamic MRI of the TMJ in order to determine the condition of the TMJ and it is found to be a reliable and precise method. Moreover, we believe that this simple mouth opener may be a useful additional device of a radiologist performing MRI examination of the TMJs.

The use of a specially designed mouth opener for TMJ imaging with high-field magnetic resonance imaging of 3 T and an appropriate protocol leads to an adequate assessment of the TMJ condition.

## Figures and Tables

**Table 1:** The presence of artifacts is assessed during examination by two experienced raters following the presented scheme: 0—no artifacts, 1—mild artifacts, 2—moderate artifacts, 3—heavy artifacts.

Types of Artifacts	Artifacts Presented	Artifacts Missing
Signal-to-noise-ratio (SNR)	1–3	0
Susceptibility	1–3	0
Signal intensity	1–3	0
Homogeneity of the field	1–3	0

**Table 2:** Frequency of TMJ disorders in magnetic resonance imaging.

Diagnosis According to MRI	N	%
**Normal TMJ**	198	49.5
**Anterior dislocation with reduction**	46	11.5
**Anterior disclocation without reduction**	18	4.5
**Posterior dislocation**	4	1
**Ostheoarthritis**	100	25
**Disc dislocation with reduction + Ostheoarthritis**	20	5
**Disc dislocation without reduction + Ostheoarthritis**	6	1.5
**Posterior + Ostheoarthritis**	8	2
**Total**	400	100

**Table 3:** Interrater reliability (radiologist–dentist).

	ICC	95% CI
SNR	0.836	0.800–0.865
Susceptibility	0.818	0.779–0.850
Signal intensity	0.835	0.799–0.865
Homogeneity of the field	0.850	0.818–0.877

Note: ICC—Intraclass correlation coefficient; 95% CI—95% confidence intervals; SNR—Signal to noise ratio.

**Table 4:** Intermethod reliability (mouth opener vs. syringe).

	ICC	95% CI
SNR	0.910	0.891–0.926
Susceptibility	0.937	0.923–0.948
Signal intensity	0.912	0.893–0.928
Homogeneity of the field	0.907	0.887–0.924

Note: ICC—Intraclass correlation coefficient; 95% CI—95% confidence intervals; SNR—Signal to noise ratio.

## Data Availability

Not Applicable.
